# Efficacy of Hepatic Resection vs. Radiofrequency Ablation for Patients With Very-Early-Stage or Early-Stage Hepatocellular Carcinoma: A Population-Based Study With Stratification by Age and Tumor Size

**DOI:** 10.3389/fonc.2019.00113

**Published:** 2019-02-26

**Authors:** Yi-Quan Jiang, Zi-Xian Wang, Yi-Nan Deng, Yang Yang, Guo-Ying Wang, Gui-Hua Chen

**Affiliations:** ^1^Department of Hepatic Surgery and Liver Transplantation Center of the Third Affiliated Hospital, Organ Transplantation Institute, Sun Yat-sen University, Guangzhou, China; ^2^Department of Minimally Invasive Interventional Therapy, Sun Yat-sen University Cancer Center, Guangzhou, China; ^3^Department of Medical Oncology, Sun Yat-Sen University Cancer Center, Guangzhou, China; ^4^State Key Laboratory of Oncology in South China, Guangzhou, China; ^5^Artificial Intelligence Laboratory of Sun Yat-Sen University Cancer Center, Guangzhou, China; ^6^Collaborative Innovation Center for Cancer Medicine, Guangzhou, China; ^7^Guangdong Key Laboratory of Liver Disease Research, The Third Affiliated Hospital of Sun Yat-sen University, Guangzhou, China

**Keywords:** hepatic resection, radiofrequency ablation, elderly, tumor size, real-world study

## Abstract

**Background:** Because of the poor health conditions of elderly patients (age >65) with very-early-stage and early-stage hepatocellular carcinoma (HCC), primary treatment via hepatic resection (HR), or radiofrequency ablation (RFA) must be considered. However, few studies have examined this issue.

**Methods:** A retrospective cohort was obtained from the Surveillance, Epidemiology, and End Results (SEER) database from 2004 to 2015. Patients were grouped by tumor size (0–20, 21–30, 31–35, and 31–50 mm) and age (>65 and ≤65). Overall survival (OS) and disease-specific survival (DSS) were assessed.

**Results:** In total, 1912 patients aged >65 and 2,784 patients aged ≤65 were analyzed after propensity score matching (PSM). For patients >65 with tumors ≤20 mm, OS and DSS did not differ significantly between the RFA and HR groups (*p* = 0.47 and *p* = 0.76, respectively). For patients with tumors measuring 21–30 mm, the HR group had better OS and a trend toward better DSS compared with the RFA group (*p* = 0.03 and *p* = 0.09, respectively). For patients with tumors measuring 31–50 mm, the HR group had better OS and DSS compared with the RFA group (*p* < 0.001 for both). For patients <65, the HR group had better OS and DSS compared with the RFA group for all tumor sizes.

**Conclusions:** For elderly patients (age >65), RFA is recommended for tumors ≤20 mm. For patients older than 65 with tumors measuring 21–50 mm and for those younger than 65 with tumors of any size, HR is the better choice.

## Introduction

Hepatocellular carcinoma (HCC), which is the most common primary malignant tumor of the liver, is considered to be the third leading cause of all cancer-related deaths and the sixth most common cancer worldwide (1, 2). Throughout the world, ~841,000 people are diagnosed with HCC, and ~782,000 people die from HCC each year (3). The age-adjusted worldwide incidence is 10.1 cases per 100,000 person-years, and its incidence is expected to increase in the future (4). The current Barcelona Clinic for Liver Cancer (BCLC) staging system classifies patients with single tumors <5 cm in size or no more than 3 tumors each <3 cm in size without major vascular invasion and metastasis as very-early-stage and early-stage disease and recommends liver transplantation (LT), hepatic resection (HR), and radiofrequency ablation (RFA) as treatment modalities. For treatment with curative intent, HR is commonly performed for patients with solitary resectable HCC and preserved liver function, whereas RFA is recommended for early multifocal HCC (each no more than 3 cm) and for single, small HCCs with dissatisfactory liver function. RFA is based on the generation of high frequency (375–500 kHz) alternating current through an electrode tip inserted into the HCC that induces a Joule effect by ionic agitation, and thus local heat, reaching a temperature of 60–100°C, which leads to tumor cell death (5). RFA is currently the most widely adopted ablation technique because it provides better disease control and outcomes than those of percutaneous ethanol injection. Patients who undergo LT have a relatively good prognosis; (6, 7) however, a shortage of donor livers limits this treatment to some extent. Therefore, HR and RFA are the most commonly performed treatments for very-early-stage and early-stage HCC (8–10).

It has long been controversial whether primary HR or RFA should be performed for this population, especially for patients >65 years old (11, 12). Several studies have compared the efficacy of RFA and HR in patients with different-sized tumors but failed to draw any conclusions. Regarding tumors measuring 0–20 mm, recent studies have suggested that RFA should be performed rather than surgery in very-early-stage HCC (single tumors <2 cm in size) (13, 14). In contrast, in a study by Liu et al. HR was reported to be preferred over RFA in this population (15). In tumors measuring 21–30 mm, HR provided a survival advantage in comparison to that of RFA in a study by Cucchetti et al. (16). Similarly, in a population-based study by Miura et al. resection of HCC ≤3 cm resulted in better long-term survival compared with that of ablation (17). However, very few studies have investigated this issue for tumors measuring 21–30 mm. In tumors measuring 31–50 mm, there is a tendency to perform HR rather than RFA; (16, 18) however, there have been conflicting results from several studies (19–22).

From another perspective, with improvements in medical care, the proportion of elderly patients with HCC has gradually increased (23, 24). Elderly people generally have poorer health conditions and more underlying diseases than younger individuals; (25) therefore, the treatment options for these patients are different from those for younger people. However, very few studies to date have investigated the efficacy of HR and RFA in elderly patients with HCC. RFA is less invasive and is associated with fewer complications, shorter hospital stays, and lower costs than those of HR (8, 9). On the other hand, HR has a low recurrence rate and a longer survival time than RFA (26, 27). Therefore, this study aimed to assess the efficacy of HR and RFA as primary treatment for HCC patients with stratification by age and tumor size.

Data regarding treatment and outcomes of patients with very-early-stage and early-stage HCC in the Surveillance, Epidemiology, and End Results (SEER) database (2017 version, a real-world database) were examined. For the first time, we compared the efficacy between HR and RFA in patients older than 65 compared with those younger than 65 with a large sample size and first subdivided the tumor size at 21–30 mm in elderly patients. After propensity score matching (PSM) with a large sample, overall survival (OS), and disease-specific survival (DSS) were compared between patients who underwent HR and patients who underwent RFA.

## Materials and Methods

A retrospective case listing was obtained from the SEER database from 2004 to 2015. The inclusion criteria were as follows: (1) the primary site of the tumor was the liver (C22.0); (2) the histologic type was HCC (ICD-0-3: 8170-8175); (3) HR (SEER code: 20–26, 30, 36–38, 50–52, 59, 60, 66, 90) or RFA (SEER code: 16) as primary treatment was conducted; (4) there was a single tumor ≤50 mm or no more than 3 tumors each ≤30 mm; and (5) patients with macroscopic vascular invasion or metastasis were excluded. Variables including age, sex, primary tumor size, tumor count, metastasis status, α-fetoprotein (AFP) level, fibrosis score (Ishak score), survival time, SEER cause-specific death classification, and vital status recode (study cut-off used) were extracted from the database. Information on details about HR and RFA procedures such as open or laparoscopic, frequency used for ablation, temperature achieved in tumors, complications after procedures and variables concerning portal hypertension, Child-pugh class, etc. were not recorded in the SEER database. The primary outcome was OS, which is defined in the SEER database as the time until death as a result of any cause. DSS, defined as the time until death attributed to HCC, was evaluated as a secondary outcome. This is a retrospective study including human participants, and we have signed the “Data-use Agreement for the SEER 1973–2015 Research Data File.”

Patients were grouped by tumor size as follows: (1) 0–20, (2) 21–30, (3) 31–50, and (4) 31–35 mm. The cut-off value of 20 mm was based on recent studies that showed that RFA was a more appropriate treatment than surgery for patients with very-early-stage HCC (single tumor <2 cm in size) (13, 14). However, a recent study also demonstrated that HR should be performed in this population (15). Concerning tumors measuring 21–30 mm, several studies preferred HR over RFA; (16, 17) nevertheless, studies with large sample sizes allowing for adequately powered subgroup analyses are needed. Therefore, the 21–30 mm group was included to investigate the efficacy of the two treatments (HR and RFA) for nodules between 2 and 3 cm in size. The 31–50 mm group was included based on reports that lesions measuring up to 50 mm can be ablated safely (22, 28). The extra 31–35 mm group was included to investigate which treatment was more appropriate when the tumor size was slightly larger than 30 mm. It should be noted that the 31–50 mm group also included the patients with tumors measuring 31–35 mm. Patients were also grouped by age (>65 and ≤65) to investigate the influence of age because a British study reported that HCC patients aged 65 and older received less or less-active treatment and had poorer survival than younger individuals (29).

We compared the characteristics of patients who received RFA and HR using the chi-squared test for categorical variables and Student's *t*-test for continuous variables. PSM was performed to maintain a balance between the RFA and HR groups (30). We calculated the propensity score using logistic regression with the variables that were potentially associated with DSS and OS or that were unbalanced between the two groups: age, sex, tumor size, tumor count, AFP level, and fibrosis. Patients were matched using a 1:1 nearest neighbor approach without replacement. A total of 4,696 patients (2,348 in each group) were selected after matching. We performed univariate analyses for all variables. Variables with *p* < 0.10 were included in the multivariate analysis. A Cox proportional hazards regression analysis was used to determine the simultaneous impact of potential confounders, including age, AFP level, fibrosis, and tumor count. The Kaplan-Meier method with a log-rank test was applied to compare the survival of patients. All statistical tests were two-sided, and *p* < 0.05 was considered statistically significant. For all analyses, we calculated hazard ratios with 95% confidence intervals (Cis). All statistical analyses were performed using SPSS 23.0 for Windows (SPSS, Chicago, IL, USA) and R software (version 3.4.4) with the rms, survival, and MatchIt packages.

## Results

### Baseline Characteristics

**Figure 1:**
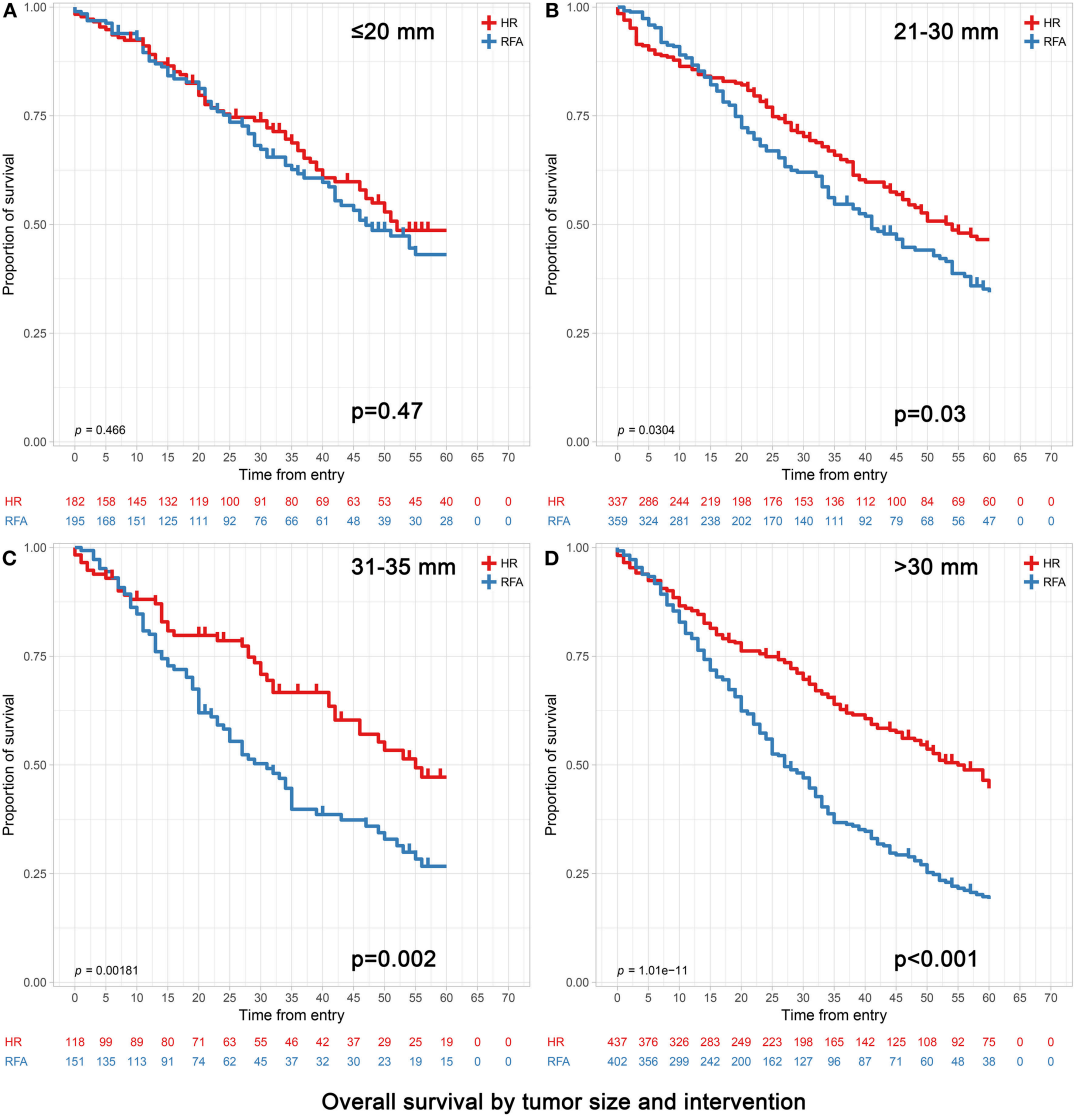
Overall survival analyses based on tumor size groups and interventions within each group (age >65). **(A)** 0–20 mm group; **(B)** 21–30 mm group; **(C)** 31–35 mm group and **(D)** 31–50 mm group. **(C)** The extra 31–35 mm group was to investigate which treatment was more appropriate when the tumor size was slightly larger than 30 mm. **(D)** The 31–50 mm group also included the patients with the 31–35 mm tumors in **(C)**.

**Figure 2:**
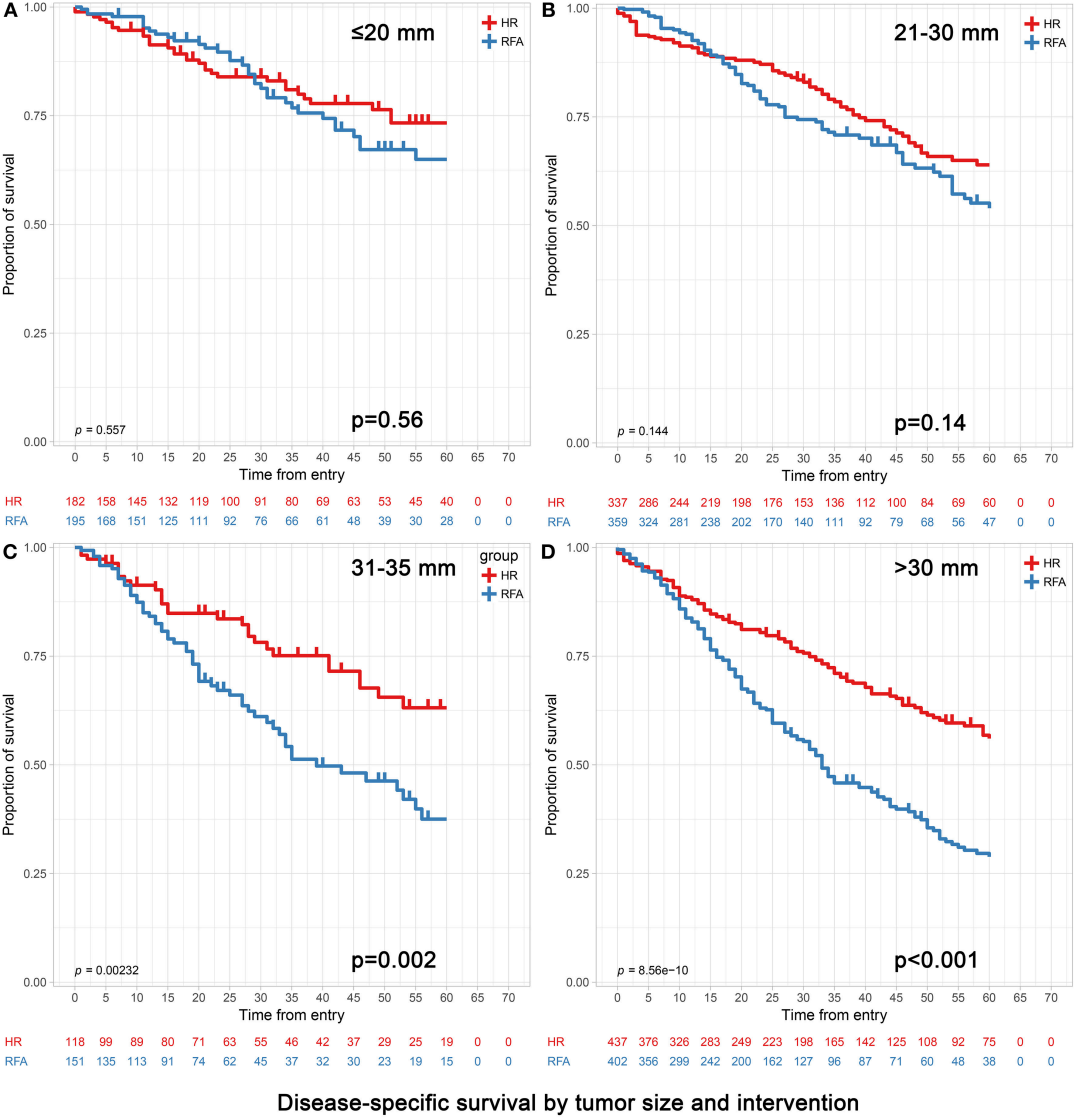
Disease-specific survival analyses based on tumor size groups and interventions within each group (age >65). **(A)** 0–20 mm group; **(B)** 21–30 mm group; **(C)** 31–35 mm group and **(D)** 31–50 mm group. **(C)** The extra 31–35 mm group was to investigate which treatment was more appropriate when the tumor size was slightly larger than 30 mm. **(D)** The 31–50 mm group also included the patients with the 31–35 mm tumors in **(C)**.

Of the 97,118 patients with a diagnosis of HCC in the SEER database during 2004–2015, a total of 6,076 patients met the inclusion criteria. The median follow up time was 28 months (interquartile range, 16–52 months). The baseline characteristics of patients before PSM and after PSM (*n* = 4,696) are presented in Tables 1, 2. Before PSM, patients in the RFA group were significantly older, had a higher proportion in the 0–30 mm tumor size, a higher level of AFP and were more likely to be classified as having cirrhosis compared to those in the HR group. After PSM, the variables in each population stratified by tumor size were approximately balanced between the RFA and HR groups (Table 2 for all patients and Table 3 for patients older than 65 stratified by tumor size). Kaplan-Meier survival analyses (Figures 1, 2) were performed based on tumor size groups (0–20, 21–30, and 31–50 mm groups and an extra specific 31–35 mm group) and interventions within each group.

**Table 1:** Baseline information for patients before PSM.

	**Age > 65**			**Age ≤65**		
**Characteristics**	**HR (*n* = 956)**	**RFA (*n* = 1,436)**	***p***	**HR (*n* = 1,392)**	**RFA (*n* = 2,291)**	***p***
**Age**						
66–75 (<50)	678	935	0.012	217	284	0.006
76–85 (51–65)	256	458		1,175	2,007	
>85	22	43				
**Sex**						
Male	634	923	0.305	1,065	1,844	0.004
Female	322	513		327	447	
**Tumor size**						
≤20 mm	182	439	<0.001	326	685	<0.001
21–30 mm	337	573		457	991	
31–50 mm	437	424		609	615	
**Tumor count**						
1	792	1,165	0.49	1,279	2,055	0.006
2	138	221		89	209	
3	26	50		24	27	
**Fibrosis**						
Severe or cirrhosis	181	423	<0.001	330	814	<0.001
None or not stated	775	1,013		1,062	1,477	
**AFP Level**						
Elevated	400	741	<0.001	712	1,359	<0.001
Normal or borderline	297	442		369	602	
Unknown	259	253		311	330	
**Type of HR**						
Segmentectomy	653			901		
Hemihepatectomy	224			297		
Extended Hemihepatectomy	79			194		

*HR, Hepatic resection; RFA, Radiofrequency ablation; AFP, α-fetoprotein; Fibrosis (Ishak score: severe or cirrhosis, 5–6; none or not stated: 0–4); PSM, Propensity score matching; Segmentectomy, resection of any one or two contiguous segments; Hemihepatectomy, resection of right or left hemiliver; Extended hemihepatectomy, right or left trisectionectomy*.

**Table 2:** Baseline information for patients after PSM.

	**Age > 65**			**Age ≤65**		
**Characteristics**	**HR (*n* = 956)**	**RFA (*n* = 956)**	***p***	**HR (*n* = 1,392)**	**RFA (*n* = 1,392)**	***p***
**Age**						
66–75 (<50)	678	664	0.63	217	204	0.49
76–85 (51–65)	256	273		1,175	1,188	
>85	22	19				
**Sex**						
Male	634	627	0.74	1,065	1,100	0.11
Female	322	329		327	292	
**Tumor size**						
≤20 mm	182	195	0.27	326	344	0.18
21–30 mm	337	359		457	487	
31–50 mm	437	402		609	561	
**Tumor count**						
1	792	787	0.81	1,279	1,289	0.26
2	138	146		89	89	
3	26	23		24	14	
**Fibrosis**						
Severe or cirrhosis	181	206	0.16	330	348	0.43
None or not stated	775	750		1,062	1,044	
**AFP level**						
Elevated	400	432	0.30	712	734	0.71
Normal or borderline	297	288		369	356	
Unknown	259	236		311	302	
**Type of HR**						
Segmentectomy	653			901		
Hemihepatectomy	224			297		
Extended Hemihepatectomy	79			194		

*HR, Hepatic resection; RFA, Radiofrequency ablation; AFP, α-fetoprotein; Fibrosis (Ishak score: severe or cirrhosis, 5–6; none or not stated: 0–4); PSM, Propensity score matching; Segmentectomy, resection of any one or two contiguous segments; Hemihepatectomy, resection of right or left hemiliver; Extended hemihepatectomy, right or left trisectionectomy*.

**Table 3:** Characteristics of patients >65 years old stratified by tumor size after PSM.

	**≤20 mm**			**21–30 mm**			**31–50 mm**		
**Characteristics**	**RFA**	**HR**	***p***	**RFA**	**HR**	***p***	**RFA**	**HR**	***p***
**Age**									
66–75	156	145	0.91	248	229	0.71	260	304	0.32
76–85	35	32		108	103		130	121	
>85	4	5		3	5		12	12	
**Sex**									
Male	102	110	0.51	228	224	0.41	271	300	0.7
Female	77	72		131	113		131	137	
**AFP level**									
Elevated	75	75	0.48	147	133	0.86	210	202	0.12
Normal or borderline	53	55		112	104		123	138	
Unknown	67	52		100	100		69	97	
**Tumor count**									
1	131	126	0.31	248	229	0.94	402	437	
2	43	45		95	93		0	0	
3	5	11		16	15		0	0	
**Fibrosis**									
None or not stated	43	42	0.81	72	58	0.34	91	81	0.14
Severe or cirrhosis	152	140		287	279		311	356	

*HR, Hepatic resection; RFA, Radiofrequency ablation; AFP, α-fetoprotein; Fibrosis (Ishak score: severe or cirrhosis, 5–6; none or not stated: 0–4); PSM, Propensity score matching*.

### Comparison After Propensity Score Matching (Age >65)

In patients with tumors ≤20 mm, RFA and HR were associated with similar survival durations. Patients achieved 3- and 5-year OS of 73.63 and 63.19% for HR and 72.82 and 64.62% for RFA, respectively (*p* = 0.47). Correspondingly, patients achieved 3- and 5-year DSS of 84.62 and 81.87% for HR and 85.64 and 82.05% for RFA, respectively (*p* = 0.56). After adjusting confounding factors affecting OS and DSS, the OS and DSS did not differ significantly between RFA and HR (OS hazard ratio, 1.13; 95% confidence interval [95% CI], 0.81–1.59, *p* = 0.47 and DSS hazard ratio, 1.08; 95% CI, 0.67–1.73, *p* = 0.76) (Tables 4, 5).

**Table 4:** Survival of patients by treatment group and tumor size (after PSM).

	**0–20 mm**			**21–30 mm**			**31–50 mm**		
**Size group%**	**RFA%**	**HR%**	***p***	**RFA%**	**HR%**	***p***	**RFA%**	**HR%**	***p***
**FOR PATIENTS AGED >65**									
3-years OS	72.82	73.63	0.47	65.46	72.70	0.03	50.25	71.17	<0.001
5-years OS	64.62	63.19		56.55	63.50		40.30	62.93	
3-years DSS	85.64	84.62	0.56	79.94	83.68	0.09	60.45	77.80	<0.001
5-years DSS	82.05	81.87		74.93	78.34		53.48	72.08	
**FOR PATIENTS AGED ≤65**									
3-years OS	74.34	79.14	0.005	69.82	78.77	<0.001	53.48	72.09	<0.001
5-years OS	66.47	72.09		61.19	70.90		46.35	64.70	
3-years DSS	83.38	88.65	0.002	82.14	85.12	0.02	63.28	77.34	<0.001
5-years DSS	77.55	84.36		76.18	79.65		57.58	71.10	

*HR, hepatic resection; RFA, radiofrequency ablation; OS, overall survival; DSS, disease-specific survival; PSM, propensity score matching*.

**Table 5:** Hazard ratios after adjusting for confounding factors (after PSM).

	**Age >65**			**Age ≤65**		
**Size group**	***p***	**Hazard ratio**	**95% CI**	***p***	**Hazard ratio**	**95% CI**
**0–20 mm**						
RFA vs. HR (OS)	0.47	1.13	0.81–1.59	**0.004**	1.51	1.14–1.99
RFA vs. HR (DSS)	0.76	1.08	0.67–1.73	**0.03**	1.34	1.03–1.65
**21–30 mm**						
RFA vs. HR (OS)	**0.03**	1.30	1.03–1.65	**<0.001**	1.59	1.27–1.98
RFA vs. HR (DSS)	0.09	1.30	0.96–1.78	**0.026**	1.37	1.04–1.80
**31–50 mm**						
RFA vs. HR (OS)	**<0.001**	2.00	1.63–2.45	**<0.001**	1.77	1.48–2.11
RFA vs. HR (DSS)	**<0.001**	2.06	1.63–2.59	**<0.001**	1.72	1.42–2.10

*HR, Hepatic resection; RFA, Radiofrequency ablation; OS, Overall survival; DSS, Disease-specific survival; PSM, Propensity score matching. Bold type indicates statistical significance*.

Among those with tumors measuring 21–30 mm, the 3- and 5-year OS were significantly better in the HR group than in the RFA group (72.70 vs. 65.46% and 63.50 vs. 56.55%, respectively; adjusted HR = 1.30, 95% CI, 1.03–1.65; *p* = 0.03). The 3- and 5-year DSS rates did not differ significantly between HR and RFA (83.68 vs. 79.94%, 78.34 vs. 74.93%; adjusted HR = 1.30, 95% CI, 0.96–1.78; *p* = 0.09) (Tables 4, 5).

In patients with tumors measuring 31–50 mm, those who underwent HR had a better prognosis than those who underwent RFA, especially in terms of survival at 5 years. Patients achieved 3- and 5-year OS of 71.17 and 62.93% and 50.25 and 40.30% for HR and RFA, respectively (*p* < 0.001). Correspondingly, they achieved 3- and 5-year DSS of 77.80 and 72.08% and 60.45 and 53.48% for HR and RFA, respectively (*p* < 0.001). After adjusting confounding factors affecting OS and DSS, the OS and DSS with RFA were found to be significantly worse than those with HR (OS hazard ratio, 2.00; 95% CI, 1.63–2.45, *p* < 0.001 and DSS hazard ratio, 2.06; 95% CI, 1.63–2.59, *p* < 0.001) (Tables 4, 5).

Notably, when the tumor size was slightly larger (i.e., in the 31–35 mm range), patients receiving HR still had significantly better OS and DSS than those receiving RFA (Table 4).

The multivariate analysis revealed that the age, tumor size, type of surgery and AFP level significantly affected OS (Table 6). Tumor size, type of surgery and AFP level also had a significant effect on DSS (Table 6).

**Table 6:** Multivariate analysis for survival in patients with age >65.

	**Overall survival**			**Disease-specific survival**		
**Variable**	**Hazard ratio**	**95% CI**	***p***	**Hazard ratio**	**95% CI**	***p***
**Age (years)**						
66–75	0.80	0.70–0.92	0.002			
76–85	Reference					
>85	1.15	0.74–1.80	0.53			
**Size**						
≤20 mm	Reference			Reference		
21–30 mm	1.11	0.92–1.34	0.29	1.34	1.03–1.74	0.03
31–50 mm	1.39	1.16–1.67	<0.001	1.66	1.30–2.12	<0.001
**Type of surgery**						
HR	Reference			Reference		
RFA	1.60	1.40–1.82	<0.001	1.67	1.42–1.97	<0.001
**AFP**						
Elevated	Reference			Reference		
Normal or borderline	0.68	0.58–0.80	<0.001	0.57	0.46–0.70	<0.001
Unknown	0.99	0.85–1.16	0.94	0.99	0.82–1.20	0.91
**Fibrosis**						
Severe or cirrhosis	Reference					
None or not stated	0.75	0.65–0.88	<0.001			

*HR, Hepatic resection; RFA, Radiofrequency ablation; AFP, α-fetoprotein; Fibrosis (Ishak score: severe or cirrhosis, 5–6; none or not stated: 0–4)*.

### Comparison After Propensity Score Matching (Age ≤65)

The baseline characteristics of all patients with age ≤65 (*n* = 3,683) and after PSM (*n* = 2,784) are also presented in Tables 1, 2. Variables were approximately balanced between the RFA and HR groups after PSM. After adjusting confounding factors affecting OS and DSS, the OS and DSS with RFA was found to be significantly worse than those with HR in all tumor size groups for patients <65 (Tables 3, 5).

### Sensitivity Analysis

For sensitivity analysis, we compared the efficacy of RFA and HR in the patient cohorts aged >65 and ≤65 before PSM, adjusted for baseline features. The results were consistent with those in the primary analyses (Table 7).

**Table 7:** Hazard ratios after adjusting for confounding factors (before PSM).

	**Age >65**			**Age ≤65**		
**Size group**	***p***	**Hazard ratio**	**95% CI**	***p***	**Hazard ratio**	**95% CI**
**0–20 mm**						
RFA vs. HR (OS)	0.28	1.17	0.88–1.56	**0.001**	1.51	1.18–1.92
RFA vs. HR (DSS)	0.52	1.15	0.76–1.73	**0.003**	1.63	1.18–2.25
**21–30 mm**						
RFA vs. HR (OS)	**0.004**	1.38	1.11–1.71	**<0.001**	1.64	1.34–2.00
RFA vs. HR (DSS)	**0.04**	1.34	1.01–1.77	**0.008**	1.40	1.09–1.77
**31–50 mm**						
RFA vs. HR (OS)	**<0.001**	2.01	1.64–2.45	**<0.001**	1.74	1.46–2.07
RFA vs. HR (DSS)	**<0.001**	2.04	1.62–2.56	**<0.001**	1.69	1.39–2.05

*HR, Hepatic resection; RFA, Radiofrequency ablation; OS, Overall survival; DSS, Disease-specific survival; PSM, Propensity score matching. Bold type indicates statistical significance*.

## Discussion

Image-guided ablation techniques have evolved considerably in the past 20 years. Among several alternate technologies, including RFA, microwave ablation, percutaneous ethanol injection, cryoablation, etc., RFA has been the most popular technique to date due to its proved efficacy and safety. Percutaneous monopolar RFA was proved to reach complete ablation (define by the absence of residual enhancement on contrast-enhanced CT or MRI imaging) of HCCs smaller than 5 cm in over 95% of cases, (31–37) thus becoming established as potentially first-line treatment option in early-stage HCC, especially in very-early-stage HCCs smaller than 2 cm. Nevertheless, RFA was still associated with worse survival outcomes and higher recurrence rates vs. HR, even in HCCs smaller than 2 cm (38). Furthermore, a study from the Beaujon group found that, unlike ablation, most recurrences after HR are within Milan criteria (39). However, several studies suggested that RFA might be associated with less morbidity and a better quality of life, (16, 20, 40) which are advantages for elderly patients. With improvements in medical care, the proportion of elderly patients with HCC has gradually increased (23, 24). Because of the unique characteristics of this population, (25) it is necessary to determine whether HR or RFA should be performed as primary treatment. In this study, we aimed to investigate this issue based on information from a real-world United States database.

In a study by Takahashi et al. the efficacy and safety of RFA were compared between older and younger patients, and the authors concluded that RFA was efficient and safe in the older patients (41). On the other hand, several studies have shown that selected older patients with HCC can also tolerate surgery with survival and postoperative complication rates similar to those of younger patients (42, 43). In most cases, elderly patients and clinicians are more likely to choose RFA than HR because elderly people generally have poorer health conditions and more underlying diseases than younger patients (25) and because RFA has the advantages of less invasiveness, fewer complications, shorter hospital stays, and lower costs compared with HR. However, there is no evidence that these patients would have a better prognosis if they chose RFA over HR. In elderly patients, the long-term outcomes, the safety and the feasibility of RFA remain unclear and should be confirmed in a prospective study.

Very few studies have investigated whether older patients with very-early-stage or early-stage HCC would benefit more from HR or RFA. In one study, Peng et al. (11) concluded that RFA had better efficacy than HR for elderly patients with HCC tumors <3 cm. However, they did not further investigate the efficacy of HR and RFA in the ≤20 and 21–30 mm groups. In a study by Yazici et al. (12) LHR was found to be tolerated as well as RFA in elderly patients (age >65) with similar comorbidities. However, the patients included in their study were not limited to those with very-early-stage or early-stage HCC. In addition, the sample sizes of the two abovementioned studies were very small, which may have led to unreliable conclusions.

In our study, OS and DSS did not differ significantly between the RFA and HR groups among elderly patients with tumor size ≤20 mm; this finding indicates that RFA can be recommended as a first-line treatment for these patients. For tumors measuring 21–30 mm, OS was worse in the RFA group compared with the HR group; in a study by Peng et al. RFA was associated with better OS and recurrence-free survival compared with HR. This discrepancy might be partially explained by the relatively small sample size in the study by Peng et al. (*n* = 63 in the RFA group and *n* = 60 in the HR group). Although the difference in DSS between the RFA and HR groups was not statistically significant, HR showed a trend toward better DSS compared with RFA, and the effect size was comparable to that for OS. Therefore, HR might be the preferred option in this patient subset. In terms of tumors measuring 31–35 mm and those measuring 31–50 mm, the OS and DSS were markedly better in the HR group than in the RFA group, indicating that this subgroup of elderly patients >65 years old can benefit from HR. We believe that HR—in particular, LHR—is the better treatment choice for this population.

Notably, in patients with tumors ≤20 mm, different results were found between patients >65 years old and those ≤65 years old, indicating that different treatment options should be used for these groups. In patients younger than 65, HR provided better OS and DSS compared with RFA, whereas for patients older than 65, RFA and HR led to similar OS and DSS. These findings are consistent with those of Nagasue et al. (44) who demonstrated that elderly patients (age >70) had a higher mortality rate and a lower long-term survival rate than those who were <50 years old. Retrospective comparisons between elderly patients >70 years old and patients <50 years old who had undergone surgery during the same period yielded the following results: a hospital mortality rate of 18.8 vs. 11.6% and a 5-year survival rate of 24.3 vs. 48.6%. Therefore, the results of our study may be explained by a decreasing long-term OS rate and increasing mortality rate with increasing age in the patients who received HR, whereas the patients who received RFA exhibited a smaller influence of age. As a result, patients who are >65 years old with tumors ≤20 mm should undergo RFA because it is more cost effective than HR (16).

There were some limitations to our study. First, to our knowledge, both tumor progression and liver function can affect OS. In the SEER database, only the extent of fibrosis (fibrosis score) was recorded; this factor is relevant to liver function and was kept balanced between the RFA and HR groups after PSM. Liver function in both populations can be considered balanced to some extent. Moreover, the DSS analysis also yielded a similar conclusion. Second, due to design limits of the SEER program, information on details about ablation procedures such as frequency used for tumor ablation, temperature achieved in the tumor, complications after RFA, provider experience and surgery procedures such as open or laparoscopic, etc. are not available in the SEER database. Because of the population-based nature of SEER program, which provides information on cancer statistics among the whole United States population, it is impractical to unify the standard of procedures among different medical centers. Despite these limitations, the use of the SEER data from the real world enables us to draw convincing conclusions on the basis of a large sample of elderly patients with very early or early-stage HCC, which is not possible in single-center studies.

In conclusion, RFA is recommended as the first-line treatment for elderly patients (age >65) with tumors ≤20 mm. In patients with tumors measuring 21–50 mm, HR is the better choice. In patients ≤65 years old, HR is recommended for all very-early-stage or early-stage HCC patients with sufficient liver function.

## Data Availability

All data and materials have been made publicly available at the SEER database and can be accessed at https://seer.cancer.gov/.

## Author Contributions

Y-QJ made primary contributions to the conception and design, acquisition of data, and analysis and interpretation of data and participated in critical drafting and revising of the article for important intellectual content. Z-XW and Y-ND made substantial contributions to conception and design and acquisition of data. G-HC, G-YW, and YY gave final approval of the version to be submitted and any revised versions.

### Conflict of Interest Statement

The authors declare that the research was conducted in the absence of any commercial or financial relationships that could be construed as a potential conflict of interest.

## Abbreviations

HCC: hepatocellular carcinoma
HR: hepatic resection
RFA: radiofrequency ablation
LT: liver transplantation
SEER, surveillance, epidemiology: and end results
BCLC: Barcelona Clinic Liver Cancer
PSM: propensity score matching
OS: overall survival
DSS: disease-specific survival
HR: hazard ratio
CI: confidence interval.
